# Prevalence of Adverse Skin Reactions in Nursing Staff Due to Personal Protective Equipment during the COVID-19 Pandemic

**DOI:** 10.3390/ijerph191912530

**Published:** 2022-10-01

**Authors:** Claudia Westermann, Nika Zielinski, Christiane Altenburg, Madeleine Dulon, Olaf Kleinmüller, Jan Felix Kersten, Albert Nienhaus

**Affiliations:** 1 Department of Occupational Medicine, Hazardous Substances and Public Health, Institution for Statutory Accident Insurance and Prevention in the Healthcare and Welfare Services, 22089 Hamburg, Germany; 2 Institute for Health Services Research in Dermatology and Nursing, University Medical Center Hamburg-Eppendorf, 20251 Hamburg, Germany

**Keywords:** healthcare worker, COVID-19, personal protective equipment (PPE), adverse skin reactions, pandemic

## Abstract

In order to prevent the nosocomial transmission of the SARS-CoV-2 virus, it has become necessary for health workers to increase their use of personal protective equipment (PPE). The aim of the study was to investigate the prevalence and influencing factors for adverse skin reactions (ASR) due to occupational PPE use among nursing staff in Germany during the COVID-19 pandemic. The study uses a mixed methods design. A focus group was created with experts from the field of healthcare, and an online survey was then carried out among nursing staff. Influencing factors were identified using multivariate logistic regression via odds ratios (ORs) with 95% confidence intervals (CIs). A total of 2274 nursing staff took part in the survey, with 1967 included in the analysis. The prevalence of ASR was 61%, with 94% affecting at least one area of the face. Statistically significant factors of influence were Filtering Face Peace (FFP) mask wearing duration of ≥4 h, a history of contact allergies, and being female and young. A pre-existing skin disease had a protective effect. The prevalence of PPE-related ASR underlines the necessity for targeted preventive measures for nursing staff during pandemic situation.

## 1. Introduction

COVID-19 infections and the spread of the pandemic pose a major challenge to healthcare systems worldwide. Healthcare workers (HCWs) are particularly susceptible to infections [1]. In order to prevent the nosocomial transmission of the SARS-CoV-2 virus, the use of personal protective equipment (PPE), particularly various types of protective masks, has become essential in all areas of healthcare [2]. There are indications in the literature that HCWs developed skin irritations during the pandemic as a result of having to wear PPE more often. Skin changes in the face and an increase in eczema on the hands had already been reported in Asia at an early stage of the pandemic, sometimes with high prevalence rates [3,4]. Reviews have found the correlation between adverse skin reactions (ASR) and HCWs wearing PPE to be a global phenomenon during the COVID-19 pandemic [5,6,7]. Influencing factors included wearing frequency and duration, the extent of the PPE required, the use of FFP masks compared to surgical masks, female sex, younger age, not using moisturisers, and existing dermatitis [5,6,7,8]. However, the number of studies reporting adjusted effect estimates is low. Ong et al. [9] reported PPE-related headache among HCWs during the COVID-19 pandemic. In the study population, wearing an N95 mask and protective goggles for over four hours per shift was associated with headache, thermal discomfort, the accumulation of moisture, and breathing difficulties [9]. When wearing a PPE, thermal factors in combination with moisture may result in a non-specific skin irritation or the worsening of congenital skin conditions [10,11]. ASR can lead to impairments and the inability to work. So far, there is little data from Germany showing the extent to which HCWs are affected by occupational PPE-related ASRs during the pandemic. In particular, PPE-related facial ASR have not been studied in depth so far. According to a prospective study, nurses are most frequently affected by occupational dermatoses compared to other health care professions [12]. Beyond the pandemic situation, this occupational group has faced high workloads due to the demographically induced increase in care-dependent people combined with the critical shortage of care workers in Germany [13]. The assumption of a high prevalence of occupational PPE-related ASRs among nursing staff due to patient-related activities during pandemic was to be investigated. The aim of the study was to investigate the prevalence of and factors correlating with ASRs in nurses due to occupational PPE use during the COVID-19 pandemic in Germany. In addition, experiences in dealing with ASR were surveyed in order to generate further actions and prevention measures.

## 2. Materials and Methods

For the purposes of this study, the term PPE is understood in accordance with its international usage and includes protective equipment used to protect oneself and others. In Germany, the use of PPE is stipulated by the Technical Rules for Biological Agents (Technische Regel für Biologische Arbeitsstoffe—TRBA 250) according to which it is primarily for use by employees for their own safety. In order to ensure this, the term PPE is associated with specific technical characteristics. These criteria are not fulfilled by a surgical mask in an occupational setting involving specific risks of infection. As a result, it is not classed as PPE by TRBA 250.

The term FFP is an abbreviation of the term “Filtering Face Piece”. These masks, FFP2 and FFP3, belong to the product category PPE. The questionnaire asked for differentiated information on the duration of use, and the data on FFP2 and FFP3 masks were entered into the model as one variable, “FFP mask”. They meet the standards for protection against droplets and aerosols and are similar to that of a N95 mask.

### 2.1. Study Design, Study Population and Statistical Analysis

In order to analyse the prevalence of new ASR developed during the pandemic, associated factors, and potential preventive measures, qualitative and quantitative methods were used (mixed methods approach). The results of this work are reported here focused on the survey. 

#### 2.1.1. Qualitative Methods

The assumption of a high occurrence of PPE-related ASR among nursing staff during the pandemic was qualitatively examined in a first step. Experts from different fields were invited to a focus group discussion in order to obtain as comprehensive a picture as possible of the situation in the health sector and to establish cooperation for the development of the questionnaire.

Factors regarding the development and prevention of occupational PPE-related ASRs should also be discussed. A total of 17 experts were invited to participate in a moderated and guideline-based focus group discussion in November 2020.

##### Statistical Analysis

The focus group was documented, transcribed [14], and systematically evaluated using a theory-led approach [15] in MAXQDA20. The evaluation was carried out by two people independently, and the interrater reliability was determined through Cohen’s Kappa coefficient [16].

#### 2.1.2. Quantitative Methods

An online survey was then carried out using a cross-sectional design. The invitation was sent to members of the German Nurses Association (Deutscher Berufsverband für Pflegeberufe—DBfK), the trade union ver.di (Vereinte Dienstleistungsgewerkschaft—ver.di), and the German Federal Association of Private Providers of Social Services (Bundesverband privater Anbieter sozialer Dienste e.V.—bpa). The prerequisite for participation was active employment in a nursing profession and participation in the period from 21 May 2021 to 8 October 2021.

##### Questionnaire

The survey was carried out anonymously using the online survey software Unipark (Tivian XI GmbH, Cologne, Germany) [17]. Little large-scale research has been conducted on the phenomenon of PPE-related (facial) ASR in an occupational setting during the pandemic. Specific validated questionnaires were therefore not available. Qualitative results were taken into account when compiling the questionnaire, and feedback was obtained continuously from occupational dermatological experts. The self-developed, standardised questionnaire used comprised 43 items and was tested for comprehension and the required time to fill it out in the pre-test for nursing staff (*n* = 11). The questionnaire was divided into the following sub-sections: The first section collected sociodemographic data (e.g., age, sex, profession, area of work, working hours, and years in the profession). The second section dealt with work-related PPE use (type and duration of use) and potential associated factors for new ASR based on earlier publications and the results of the focus group (such as existing skin conditions, allergies, and PPE availability). The third part asked participants about new ASR (e.g., localisation, symptoms, duration of symptoms, potential PPE trigger, diagnosis, and extent (mild, moderate, and severe)). Finally, there were some questions about potential preventive measures used by the participants in order to prevent new ASR or prevent existing symptoms from worsening.

##### Statistical Analysis

An a priori sample size calculation was conducted based on the publication by Kiely et al. [17], assuming the prevalence of a new ASR on the cheek area of nursing staff as a result of PPE use at 12% ± 2%, with a probability of alpha error of 5%. The calculation was carried out using the OpenEpi version 2.3. A total sample size of at least 1024 participants was required for a sufficient power of 80%.

The data from the online survey were evaluated descriptively (absolute number, relative frequency, mean and standard deviation [SD]). Bivariate analyses were conducted in order to investigate potential factors influencing new ASR on the face. All variables that proved statistically significant in bivariate analyses were tested for multi-collinearity and interactions. Multi-collinearity is assumed with a variance inflation factor (VIF) of over 10 or a tolerance below 0.1. Interactions are deemed statistically significant if the *p*-value of the interaction term is lower than 0.05. Finally, a binary logistic regression analysis was carried out. All statistical analyses were carried out as part of a complete case analysis (CCA). Participants with incomplete information for the variables used in the binary logistic regression model were excluded from CCA. We performed forward selection to include the independent variables that had the highest correlation with the dependent variable ‘new ASR’. Adjusted effect estimates are reported as odds ratios (OR) and 95% confidence intervals (CI). The significance level was set at 0.05. The goodness of fit for the model was evaluated using the Hosmer–Lemeshow test. Goodness of fit is acceptable when the *p*-value is higher than the significance level. The statistical analyses were carried out using SPSS version 27.0 (Armonk, NY, USA: IBM Corp.).

### 2.2. Ethics

Participation in the focus group was voluntary and dependent on consenting to the anonymous collection and processing of data. The survey participants were informed that the data were collected and evaluated anonymously without personal disclosures. Participation was voluntary and dependent on consent to data processing being provided. The answering process could be terminated at any time. The data collected are not subject to the General Data Protection Regulation (Datenschutzgrundverordnung—DSGVO) for the processing of personal data. The data protection concept was developed in consultation with the data protection officer from the Statutory Accident Insurance and Prevention in the Health and Welfare Services (BGW). A vote is not required from an ethics committee for data collected anonymously.

## 3. Results

### 3.1. Focus Group Discussion with Experts

The interrater reliability of the two independently evaluating persons was 0.8, indicating high agreement. A total of ten experts from the fields of academic occupational dermatology, clinical care, and outpatient geriatric care, as well as representatives of outpatient and inpatient physicians and dentists, accident insurance providers, and the Federal Institute for Drugs and Medical Devices (Bundesinstitut für Arzneimittel und Medizinprodukte—BfArM) took part in the focus group discussion.

Occupational dermatologists have assumed both an increase in hand eczema and an increased incidence of facial dermatoses during the pandemic, although the expected accumulations did not appear in the consultations. In the hospital sector, in outpatient geriatric care, as well as in a specifically established Corona occupational eczema consultation, mainly cases of facial dermatoses were noticed. Nurses were particularly affected. The main risk factor for ASR caused by wearing protective masks was an pre-existing dermatological condition, while triggering factors were the frequency and duration of wear, as well as the lack of availability of suitable products during the pandemic. The experts described dermatological conditions affecting the face as causing subjective discomfort with primarily moderate symptoms. It was reported that those affected required more information with regard to handling new occupational PPE-related ASR.

### 3.2. Online Survey

**Figure 1:**
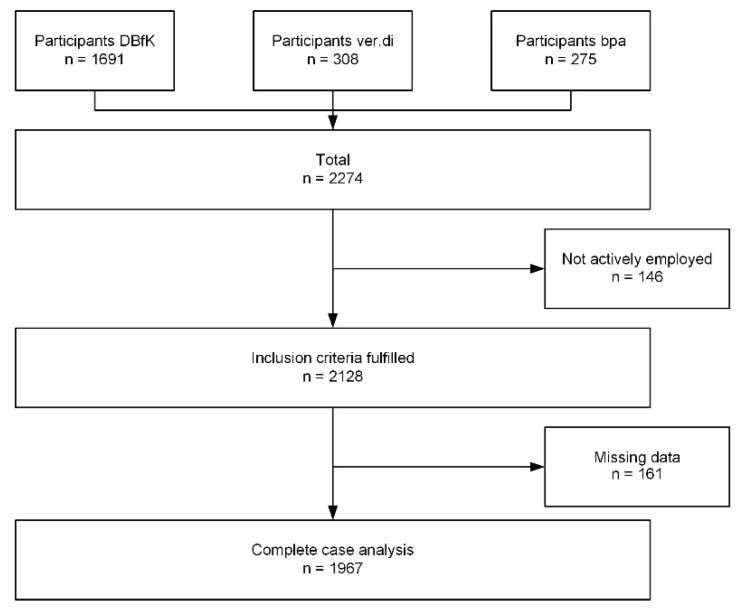
Flowchart of the complete case analysis study population.

A total of 2274 people engaged in a nursing profession, from three nationwide represented associations, took part in the online survey (DBfK *n* = 1691, bpa *n* = 275, ver.di *n* = 308). Respondents who were not actively employed (*n* = 146) and people with incomplete data with regard to the model’s variables were excluded from the analyses below (*n* = 161). The CCA included data entries from 1967 nursing staff (Figure 1).

The sample was primarily female, with an average age of 45 years (SD 12). Most of the surveyed nursing staff worked at least five days a week (70%) and were pre-dominantly employed full-time in hospitals (Table 1). Of the nurses working in a hospital, almost half worked on wards, 27% on an intensive care unit, and 20% in other settings. Of the employees caring for patients with COVID-19, one third (39%) worked in wards, and two thirds (77%) worked in intensive care units (not shown in the table).

#### 3.2.1. Pre-Existing Skin Conditions

**Figure 2:**
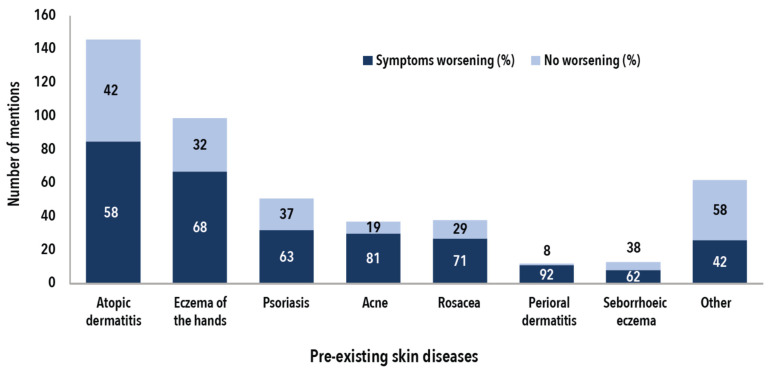
Pre-existing conditions by symptoms and percentage of cases worsened by the use of PPE in the period from January 2020 until the time of the survey (*n* = 354, multiple answers possible).

The prevalence of pre-existing skin conditions in the sample analysed was 18% (Table 1). The most common conditions mentioned were atopic dermatitis, eczema of the hands, and psoriasis (Figure 2). Of those people with pre-existing conditions, 59% stated that wearing PPE during the pandemic worsened their conditions. People with perioral dermatitis (92%), acne (81%), and rosacea (71%) were most commonly affected. Symptoms were more likely to worsen in the face area (69%) than on the hands (59%) or other areas (21%) (not shown in table).

#### 3.2.2. Prevalence of Allergies

The most common allergies in the sample analysed were hay fever (29%), contact allergy (22%), and allergic asthma (12%). Individual cases of allergies to specific foods, medicines, dust, and latex were also stated.

#### 3.2.3. Prevalence of ASR Observed for the First Time

The prevalence of ASR observed for the first time during the pandemic was 61% in the sample analysed. The majority of respondents reported having developed at least one new ASR on the face (94%, Table 2). The most commonly affected areas were around the mouth and nose (55% each), followed by the chin (50%). On average, three areas of the face (SD 2) were affected, with the mouth, nose, and chin the most frequent combination. In 14% of cases, only one area of the skin was affected. For those with new ASR in the face area, 96% stated there was a correlation with wearing FFP masks (not shown in the table). Of those with a new ASR, hands were also affected in 37% and wrists in 9%.

**Figure 3:**
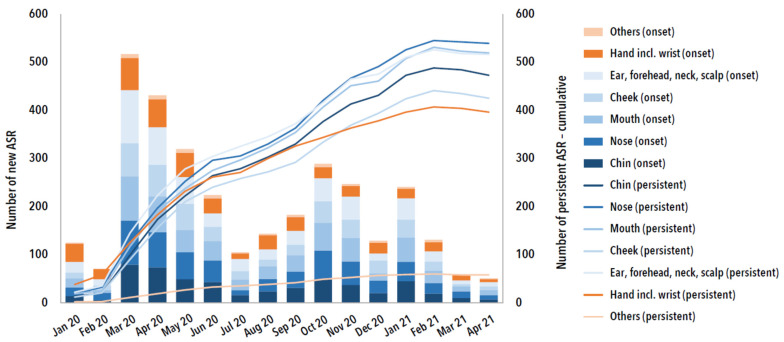
New adverse skin reaction (ASR) and their proportion (cumulative) with persistent complaints grouped by skin area over time from January 2020 to April 2021 (*n* = 1204, multiple answers possible).

Figure 3 shows the new ASRs over time by area of skin affected for the time period until April 2021. On monthly basis, most new ASRs occurred in the period from March 2020 to April 2020. The complaints lasted a median of 10 months per skin area. At the time of the survey, the new ASR were predominantly described as persistent.

Of those with new ASR, over half reported that the PPE available was not the same as the standard product used prior to the pandemic. Two thirds stated that they did not tolerate the new PPE as well as the standard product.

#### 3.2.4. Complaints

**Figure 4:**
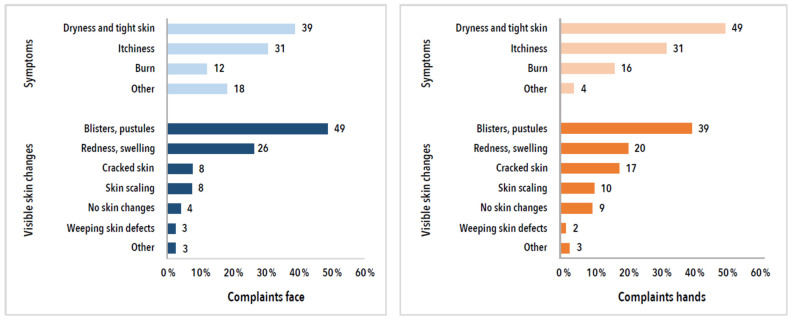
New adverse skin reactions, categorised by symptoms and visible skin changes, and stratified by area affected (face and hands, incl. wrists, *n* = 1204).

Figure 4 shows the complaints for the face and hands areas (including the wrists), categorised by symptoms and visible skin changes. Dryness and tight skin were the main symptoms both in the face and on the hands. Blistering, the development of pustules, skin redness, and swelling were the main visible changes reported in the face. For the hand area, cracked skin, redness, swelling, and skin scaling were the main changes (Figure 4).

Mild (44%) to moderate (50%) complaints in the face were most commonly reported, with only 5% of those affected reporting severe complaints at the time of the survey. Similar figures were observed for the hand area: 35% stated they had mild symptoms, 56% had moderate symptoms, and 8% had severe symptoms at the time of the survey. Only few respondents consulted a doctor (*n* = 205, 17%), most commonly a dermatologist, followed by a general and occupational medicine specialist. A new diagnosis was made in half of these cases (102 of 205, 50%). Few cases resulted in an incapacity to work (28 of 205, 14%).

#### 3.2.5. Factors Influencing New ASRs on the Face

Bivariate analyses were initially carried out in order to identify potential factors influencing the occurrence of new ASR on the face (Table S1). With regard to the mask type (surgical vs. FFP) and wearing duration, only the wearing of FFP masks for ≥4 h had a statistically significant impact on the outcome (OR 1.4 [CI 1.1–1.8; *p* = 0.008]). Other potential risk factors included a pre-existing dermatological condition and/or a contact allergy. In a comparison between the sexes, women were significantly more likely to be affected by a new ASR (61% vs. 42%). Age was only a significant factor among women. There was an interaction between age and sex, with young female nursing staff most commonly affected (OR 4.1 [CI 2.7–6.4; *p* < 0.001]). Neither the area of work, training on how to use PPE, years of professional experience, nor the type of employment had a significant impact on the occurrence of new facial ASR in the sample analysed.

**Figure 5:**
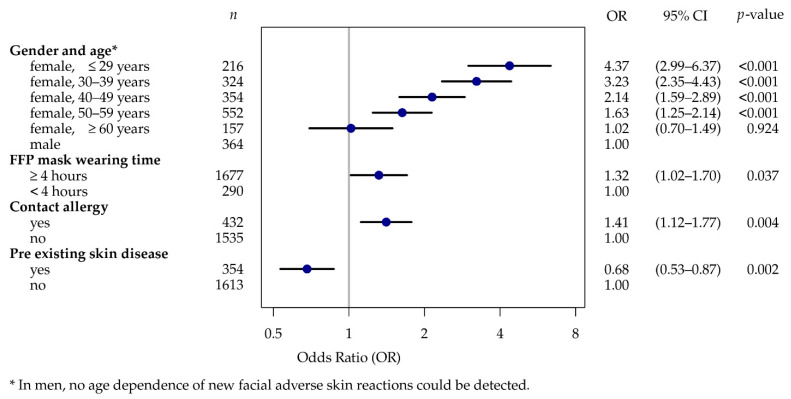
Multiple logistic regression—predictive factors for new facial ASR (*n* = 1967).

In the multiple logistic regression model with new facial ASR as the outcome variable, age, sex, FFP wearing duration, known contact allergy, and pre-existing skin condition were used as independent variables and controlled for each other (Figure 5). There was no multicollinearity (VIF < 2, tolerance > 0.95), and there was no evidence of a poor fit of the final model (*p* = 0.996). FFP wearing times of ≥4 h as compared with shorter wearing duration and a known contact allergy were associated with significantly higher risks of new facial ASR (OR 1.3 [CI 1.0–1.7; *p* = 0.037] and OR 1.4 [CI 1.1–1.8]; *p* = 0.004). The presence of a pre-existing skin disease had a protective effect in the cohort (OR 0.7 [CI 0.5–0.9; *p* = 0.002]). In comparison with men, women aged ≤29 years were 4.4 times [CI 3.0–6.4; *p* < 0.001] more likely to develop a new ASR on the face. Those aged 30–39 were 3.2 times [CI 2.4–4.4; *p* < 0.001], 40–49 year olds were 2.1 times [CI 1.6–2.9; *p* < 0.001], and the 50–59 age group was 1.6 times [CI 1.3–2.1; *p* < 0.001] more likely to develop a new facial ASR. Men and women aged over 59 years did not differ in their likelihood of developing a new facial ASR.

### 3.3. Preventive Approaches to Wearing Masks

This section first presents the results of the qualitative analysis of the focus group discussion (experts) regarding preventive measures and then those of the survey (nursing staff).

#### 3.3.1. Results from Experts Focus Group Discussion

The focus group discussed various measures to help avoid ASR caused by wearing protective masks. These measures are summarised in changing the mask if it becomes wet and keeping to wearing times and breaks. If possible, activities with and without protective masks should alternate. A selection of masks of the same protection class should be made available and a model according to compatibility and fit should be chosen. Gentle cleansing of the face with pH-neutral, fragrance-free products is advisable, and wearing make-up during working hours should be avoided. In case of new dermatoses, consultation of a doctor at an early stage is advisable; in case of existing skin diseases, treatment should be continued.

#### 3.3.2. Results from Survey

The online survey asked about measures for avoiding skin conditions affecting the face associated with mask-wearing. More than half of the HCWs gave information on this. The most frequent response was to change masks regularly. This included changing them when they became moist as well as switching from FFP masks to surgical ones, for example, for office work or other tasks without patient contact. Changing masks to one that was more tolerable was also deemed very important. There were reports that the provision of different products in a sufficient quantity from different manufacturers (selection of mask sizes, types, and models) was a problem during the pandemic. Maintaining wearing and break times was also frequently cited. However, they could not always be maintained as a result of understaffing and the enormous workload. Breaks of several days off work were associated with positive effects on the affected areas of the face. Many referred to skin care, which took more time than before workplace mask-wearing became mandatory. Only very few respondents mentioned going without make-up during working hours or consulting a doctor. In addition to the sufficient provision of different mask types, models, and sizes, the provision of skincare products and materials to relieve pressure (e.g., hydrocolloid plasters) at the workplace was also suggested.

## 4. Discussion

In the sample studied, 61% of caregivers developed an ASR during the pandemic. In 94% of these cases, at least one area of the face was affected. Symptoms were primarily described as moderate. Only few respondents consulted a doctor about their symptoms. In the multivariate regression model, female sex combined with a younger age bracket (<60 years), a contact allergy, and FFP mask-wearing time of ≥4 h were the main predictors for a statistically significant increase in the occurrence of new facial ASR during the pandemic. Young female nurses were most commonly affected, with an OR between 4.4 for nurses aged under 29 years and 1.6 for nurses aged between 50 and 59. In contrast to the results from the expert focus group discussion, an existing skin condition was shown to have a protective effect on developing new facial ASR. For some skin conditions, such as perioral dermatitis, acne, and rosacea, nursing staff reported PPE-related worsening of the existing symptoms during the COVID-19 pandemic, partly because the usual standard product was not available.

Other survey results from Germany indicate similar results, although the lack of consideration of further independent variables in the analysis should be taken into account. In their study at the LMU Hospital Munich, Niesert et al. [18] evaluated 550 participants (*n* = 80 health workers, *n* = 470 patients and/or visitors) with regard to the influence of mask-wearing duration on the prevalence of skin conditions affecting the face. HCWs showed a median daily mask-wearing duration of 4.3 h (±1.2) and a prevalence of ASR affecting the face of 49%. By comparison, non-HCWs had a lower prevalence of 7.3% with mask-wearing duration of 1.8 h (±1.2). Men and participants aged over 59 were significantly less affected. In addition to surgical and FFP masks, cloth masks (community masks) were also included in the analysis and the daily mask-wearing duration was not restricted to occupational exposure alone. Participants with a type IV hypersensitivity reaction, which could also include contact allergies, were significantly more likely to experience ASR affecting the face than people without such reactions (26% vs. 11%). The most common symptoms stated were itching, reddening, and pimples. Blistering or pustules and red skin were most commonly reported as visible skin complains in our study. The main symptoms were dryness and itching.

Guertler et al. [19] reported on the prevalence of eczema of the hands among HCWs during the COVID-19 pandemic. This study was also based on a survey at the LMU Hospital Munich. The survey revealed a significant increase in hand-washing, disinfection, and the use of hand cream among all respondents during the pandemic, regardless of whether or not they had direct contact with COVID-19 patients. A good 90% of respondents reported symptoms (such as dryness, redness, and itching) connected with acute dermatitis of the hands. In our study, regardless of the area in which they worked, 23% (443 of 1967) of respondents developed symptoms on their hands during the pandemic and 6% (110 of 1967) developed symptoms on their wrists. The increased hygiene measures such as hand-washing and disinfection did not appear to be as problematic for nursing staff as the occupational use of PPE. Wearing an FFP mask for four hours or over was a main predictive factor for the significantly higher occurrence of facial ASR, regardless of whether or not the staff were responsible for the (intensive) care of COVID-19 patients. In line with the international literature, occupational exposure to FFP masks is correlated with the development of facial ASR [5,7]. Among HCWs, nurses were the most commonly affected occupational group [5]. Other predictive factors identified in meta-analyses were female sex, younger age, and pre-existing skin conditions [5,7]. Among HCWs, nurses were the most commonly affected occupational group [5]. In our analyses, pre-existing skin conditions were shown to be protective factors in relation to the development of occupational PPE-related facial ASRs during the pandemic.

Regarding female sex and younger age as risk factors for increased development of ASRs, findings of surveillance of occupational skin diseases in the U.K. also report an association of female gender and young age in relation to occupationally acquired skin diseases [20]. As reported by Chen et al. [21], gender differences in medicine occur in the anatomy, physiology, epidemiology, and manifestations of various diseases. Skin diseases show, for example, in atopic eczema, that young women have a significantly higher prevalence than men during the reproductive years. Gender differences may alternate in the same disease at different ages and have also been observed in disease prognosis. The aetiology and pathogenesis of gender differences in the development of skin diseases are not fully understood. Conditions such as gender-specific differences in the structure and function of the skin, hormonal influences, genetic predisposition, as well as socio-cultural background and geographical or environmental factors must be taken into account [21]. Nevertheless, a healthy worker effect cannot be ruled out in the sample studied. Nurses with skin problems might tend to leave the profession; however, no correlation between age and male nurses is shown in the sample studied, which also argues against younger nurses not being sufficiently educated about wearing PPE compared to older ones. Additionally, in our bivariate analyses, training on how to use PPE and years of professional experience were not statistically significant predictors of new facial ASR.

According to Keng et al. [6] PPE-related facial ASR include mask-related acne and skin lesions caused by pressure [6]. Skin lesions may initially occur as harmless temporary erythema or pressure sores. If sufficient protective measures are not taken to care for the affected areas, the skin may crack, erode, blister, or form ulcers. The areas of the face most sensitive to pressure are the bridge of the nose and the cheeks. Skin maceration and abrasions may affect the skin’s protective barrier and lead to a secondary infection requiring medical treatment [6].

The protective effect of an FFP mask largely depends on how well it fits the contours of the wearer’s face. These masks have to fit the face very closely in order to function properly. The right fit is therefore a major preventive measure. FFP masks that fit well can minimise the risk of injuries due to pressure. Numerous studies have found that pressure injuries on the face can be traced back to the use of FFP masks or protective goggles that fit (too) tightly [6]. Hydrocolloid plasters or strips of polyurethane foam may, when used properly, help to relieve pressure on the affected areas, but this risk impeding the efficacy of the FFP mask [2,6,22]. The provision of proper alternative products with a sufficiently wide selection (model and size) and in sufficient quantity is therefore of major significance in order to avoid new facial ASR and to protect against the transmission of pathogens. The employees in our study recommended switching from FFP masks to surgical masks as a means of avoiding ASR when working in an office or in other roles without patient contact. At the time of the study, it was mandatory for HCWs in hospitals and clinics and in geriatric care in Germany to wear an FFP mask.

Both the experts and the nursing staff in the survey mentioned regularly changing masks and taking breaks from mask-wearing as a key preventive measure. This helps to minimise the friction and pressure on the face. Taking several days off at once also gives the skin the chance to recuperate. They deemed good skin care and consulting a doctor in good time as just as important. Due to the enormous workload and the long wearing duration of FFP masks as a result, good skin care is very important [22], ideally after consulting a dermatologist as quickly as possible. HCWs are confronted with a wide range of work-related stressors during the COVID-19 pandemic, which means that early warning signs from the skin, such as in the form of mild erythema, can easily be overlooked, particularly in employees with no history of dermatological conditions [6]. The early consultation of a dermatologist is also recommended according to the experiences of other occupational dermatologists [23,24]. Information about dealing with ASR and the process for Statutory Accident Insurance dermatologists could be specifically communicated to employees by company doctors, for example. Furthermore, information media and telemedicine could be useful in reaching employees and facilitating medical consultations with minimal time required. In some disciplines and patient groups, studies have shown that telemedicine does not impede the quality of care. It can keep waiting times down and improve patient satisfaction. The use of telemedicine services for dermatology has increased during the current pandemic [6]. The provision of pH-neutral moisturisers that are suitable for sensitive areas of facial skin where PPE must be worn is welcomed by employees and recommended in studies [6].

The limitations of the study design have to be taken into account when interpreting the results of this study. We assumed that nurses were exposed to PPE-related ASRs, but at the time of the study, which was planned in 2020, there was still no national data available. With the help of the experts, we identified important aspects concerning PPE-related ASRs during the pandemic and included them into the survey questionnaire, in addition to the results from international literature. The presented analyses are based on self-reporting by the nurses. Our focus was on occupational exposure; wearing times of PPE outside work were not surveyed. Montero-Vilchez et al. [7] considered this aspect in their review question; the incidence of ASRs associated with masks and gloves was almost twice as high in HCWs as in non-HCWs, which can be explained by longer wearing times for HCWs [7,18]. Furthermore, a selection bias cannot be ruled out: Employees with PPE-related ASR may have been more likely to participate in the survey than employees who were unaffected. The results presented here are mainly based on a cross-sectional survey. Causal relationships cannot be deduced without further studies with several observation periods. Online surveys also have some limitations, e.g., limited internet access, lack of privacy when filling out the questionnaire or shared computers at the workplace. These limitations cannot be completely excluded; however, they are not expected to have affected the data collection, as participants were free to choose the time, place, and hardware for completing the survey. Some of the strengths of the study include the large cross-regional sample, the differentiated data collection according to the research question and the reported adjusted effect estimators.

## 5. Conclusions

The COVID-19 pandemic has posed many challenges to the health sector, especially for nurses who perform patient-related activities with high workloads and high protection requirements for themselves and others in times of pandemic. Timely findings are important to identify problem areas and possible solutions. During the COVID-19 pandemic, nursing staff were particularly affected by dermatological conditions of the face in Germany. Wearing FFP masks for long periods, as stated in the sample analysed, is associated with developing facial ASR, regardless of whether the nurse cares for COVID-19 patients or not. Furthermore, female sex in conjunction with being aged under 59 and having contact allergy were predictors for developing new facial ASR. Knowledge of how to deal with skin diseases seems to be a protective factor. Preventive measures refer to both the behavioural and ratio level, whereby not only employers but also policy makers are called upon to improve the working conditions of nursing staff. In addition to reducing mask wearing times and ensuring regular mask breaks, providing adequate PPE in sufficient numbers is an important measure to ensure fitness to avoid facial ASR. Furthermore, low-threshold occupational health services are needed to provide fast help and advice in case of ASRs. In terms of the behavioural level, raising awareness of the risks of developing PPE-related ASR and how to handle them is an important aspect. In addition to a mild, ideally pH-neutral skin care regimen, consulting a dermatologist at an early stage is recommended. The prevalence of PPE-related ASR underlines the necessity for targeted preventive measures for affected occupational groups during a pandemic. Multi-measurement studies are required to investigate the causal relationships in the development of PPE-related ASR also under pandemic conditions.

## Figures and Tables

**Table 1:** Sociodemographic description of study population (*n* = 1967).

Characteristic (Missing Values)	Average	SD
Age (0)	45.0	11.7
**Characteristic (Missing Values)**	**Number**	**%**
Sex (0)		
Female	1603	81.5
Male	364	18.5
Profession (9)		
Healthcare and nursing specialist/assistant	1421	72.6
Geriatric nursing specialist/assistant	391	20.0
Other	146	7.5
Sector (2)		
Hospital	1184	60.2
Inpatient geriatric care	303	15.4
Outpatient geriatric care	301	15.3
Other	177	9.0
Employment type (5)		
Full time	1126	57.4
Part time	836	42.6
Working days per week (19)		
1 Day	20	1.0
2 Days	79	4.1
3 Days	177	9.1
4 Days	308	15.8
5 Days	1067	54.8
6 Days	297	15.2
Pre-existing skin disease (0)		
Yes	354	18.0
No	1613	82.0
Adverse skin reaction (0)		
Yes	1204	61.2
No	763	38.8

SD = Standard deviation % = relevant percentage.

**Table 2:** New adverse skin reactions during the COVID-19 pandemic, differentiated by skin area and number of positive responses (*n* = 1204).

	Number	%
Area of skin affected ^1^		
Mouth	666	55.3
Nose	665	55.2
Chin	601	49.9
Cheeks	542	45.0
Hands	443	36.8
Ears	427	35.5
Forehead	146	12.1
Wrists	110	9.1
Scalp	66	5.5
Neck	32	2.7
Other ^2^	92	7.6
Number of areas of skin affected		
One	162	13.5
Two	299	24.9
Three	272	22.7
Four	254	21.2
Five	120	10.0
≥Six	92	8.4
New adverse skin reaction on the face ^2^		
Yes	1131	93.9
No	73	6.1

^1^ Multiple answers permitted. ^2^ Including mouth, nose, chin, cheeks, ears, forehead, scalp, nape of the neck, and periocular region.

## Data Availability

The data are available from the corresponding author upon request.

## Supplementary Materials

The following supporting information can be downloaded at https://www.mdpi.com/article/10.3390/ijerph191912530/s1, Table S1: Bivariate analyses—factors influencing new facial ASR (*n* = 1967).
