# Supplementary material for: Prevalence of Adverse Skin Reactions in Nursing Staff Due to Personal Protective Equipment during the COVID-19 Pandemic
Source: Int J Environ Res Public Health. 2022 Oct 1;19(19):12530. doi: 10.3390/ijerph191912530 (PMC9566727; doi:10.3390/ijerph191912530)
Supplement: Supplementary file 1 [file ijerph-19-12530-s001.zip › ijerph-1935179-supplementary.pdf]

Table S1. Bivariate analyses – factors influencing new facial ASR (n = 1967)

Variable [missing value]	New ASR		No ASR		OR [95% CI]	p-value
	n	%	n	%		
**FFP mask wearing time**						
≥ 4 hours	985	58.7	692	41.3	1.4 [1.1; 1.8]	0.008
< 4 hours	146	50.3	146	49.7	1	-
**Surgical mask wearing time (1295)**						
≥ 4 hours	252	59.3	173	40.7	1.1 [0.8; 1.5]	0.575
< 4 hours	141	57.1	106	42.9	1	-
**Years in profession (4)**						
< 1 year	25	62.5	15	37.5	1.5 [0.8; 2.9]	0,211
≥ 1 year - < 20 years	572	63.1	335	36.9	1.6 [1.3; 1.9]	<0.001
≥ 20 years	532	52.4	484	47.6	1	-
**Area at work (2)**						
Hospital	694	58.6	490	41.4	1.2 [0.9; 1.5]	0.277
Inpatient geriatric care	173	57.1	130	42.9	1.1 [0.8; 1.5]	0.630
Outpatient geriatric care	166	55.1	135	44.9	1	-
Other	98	55.4	79	44.6	1.0 [0.7; 1.5]	0.963
**Employment type (5)**						
Full time	665	59.1	461	40.9	1.2 [1.0; 1.4]	0.128
Part time	465	55.6	371	44.4	1	-
**Contact allergy**						
Yes	266	61.6	166	38.4	1.2 [1.0; 1.5]	0.053
No	865	56.4	670	43.6	1	-
**Pre-existing skin disease**						
Yes	184	52.0	170	48.0	0.8 [0.6; 1.0]	0.021
No	947	58.7	666	41.3	1	-
**Age**						
≤29 years	178	68.2	83	31.8	2.9 [2.0; 4.3]	<0.001
30-39 years	276	65.9	143	34.1	2.6 [1.8; 3.7]	<0.001
40-49 years	256	57.7	188	42.3	1.9 [1.3; 2.6]	0.001
50-59 years	343	52.0	316	48.0	1.5 [1.1; 2.1]	0.021
≥ 60 years	78	42.4	106	57.6	1	-
**Sex**						
Female	978	61.0	625	39.0	2.2 [1.7; 2.7]	<0.001
Male	153	42.0	211	58.0	1	-
**Gender and age (male)**						
≤29 years	14	31.1	31	68.9	0.8 [0.3; 2.1]	0.606
30-39 years	49	51.6	46	48.4	1.8 [0.8; 4.4]	0.185
40-49 years	40	44.4	50	55.6	1.4 [0.6; 3.3]	0.496
50-59 years	40	37.4	67	62.6	1.0 [0.4; 2.4]	0.973
≥ 60 years	10	37.0	17	63.0	1	-
**Gender and age (female)**						
≤29 years	164	75.9	52	24.1	4.1 [2.7; 6.4]	<0.001
30-39 years	227	70.1	97	29.9	3.1 [2.1; 4.6]	<0.001
40-49 years	216	61.0	138	39.0	2.1 [1.4; 3.0]	<0.001
50-59 years	303	54.9	249	45.1	1.6 [1.1; 2.3]	0.011
≥ 60 years	68	43.3	89	56.7	1	-
